# Crystal structures of photosystem II from a cyanobacterium expressing *psbA*_*2*_ in comparison to *psbA*_*3*_ reveal differences in the D1 subunit

**DOI:** 10.1016/j.jbc.2022.102668

**Published:** 2022-11-02

**Authors:** Yoshiki Nakajima, Natsumi Ugai-Amo, Naoki Tone, Akiko Nakagawa, Masako Iwai, Masahiko Ikeuchi, Miwa Sugiura, Michihiro Suga, Jian-Ren Shen

**Affiliations:** 1 Research Institute for Interdisciplinary Science, Okayama University, Okayama, Japan; 2 Graduate School of Natural Science and Technology, Okayama University, Okayama, Japan; 3 Proteo-Science Research Center, Ehime University, Matsuyama, Japan; 4 Graduate School and College of Arts and Sciences, The University of Tokyo, Meguro-ku, Tokyo, Japan

**Keywords:** photosystem II, D1 protein, *psbA* gene, crystal structure, cyanobacteria, OEC, oxygen-evolving complex, PDB, Protein Data Bank, SQDG, sulfoquinovosyl diacylglycerol, TMH, transmembrane helix

## Abstract

Three *psbA* genes (*psbA*_*1*_, *psbA*_*2*_, and *psbA*_*3*_) encoding the D1 subunit of photosystem II (PSII) are present in the thermophilic cyanobacterium *Thermosynechococcus elongatus* and are expressed differently in response to changes in the growth environment. To clarify the functional differences of the D1 protein expressed from these *psbA* genes, PSII dimers from two strains, each expressing only one *psbA* gene (*psbA*_*2*_ or *psbA*_*3*_), were crystallized, and we analyzed their structures at resolutions comparable to previously studied PsbA1-PSII. Our results showed that the hydrogen bond between pheophytin/D1 (Pheo_D1_) and D1-130 became stronger in PsbA2- and PsbA3-PSII due to change of Gln to Glu, which partially explains the increase in the redox potential of Pheo_D1_ observed in PsbA3. In PsbA2, one hydrogen bond was lost in Pheo_D1_ due to the change of D1-Y147F, which may explain the decrease in stability of Pheo_D1_ in PsbA2. Two water molecules in the Cl-1 channel were lost in PsbA2 due to the change of D1-P173M, leading to the narrowing of the channel, which may explain the lower efficiency of the S-state transition beyond S_2_ in PsbA2-PSII. In PsbA3-PSII, a hydrogen bond between D1-Ser270 and a sulfoquinovosyl-diacylglycerol molecule near Q_B_ disappeared due to the change of D1-Ser270 in PsbA1 and PsbA2 to D1-Ala270. This may result in an easier exchange of bound Q_B_ with free plastoquinone, hence an enhancement of oxygen evolution in PsbA3-PSII due to its high Q_B_ exchange efficiency. These results provide a structural basis for further functional examination of the three PsbA variants.

Photosystem II (PSII) is primarily a dimeric membrane protein complex located in thylakoid membranes of various cyanobacteria, algae, and green plants and functions to split water and evolve molecular oxygen in photosynthesis. The crystal structure of a cyanobacterial PSII dimeric complex solved at 1.9 Å resolution shows that each PSII monomer consists of 17 transmembrane subunits, three extramembrane subunits, 35 chlorophylls (Chl) *a*, two pheophytins (Pheo), 12 carotenoids, two plastoquinones, one Mn_4_CaO_5_ cluster, two heme, one nonheme iron, one bicarbonate, two chlorides, and 25 lipids (1, 2). A photon absorbed by Chls of core antennae subunits, CP43 and CP47, is transferred to the reaction center Chls, Chl_D1_/Chl_D2_, or P_D1_/P_D2_, known as P680 bound to the D1 and D2 subunits, respectively, resulting in excitation of P680 to P680^+•^. P680^+•^ subsequently oxidizes D1-Tyr161, known as Tyr_Z_ (or Y_Z_), and the oxidized Tyr_Z_ extracts an electron from the Mn_4_O_5_Ca cluster at the oxygen-evolving complex (OEC). Following four consecutive electron transfer reactions, two water molecules are split into four electrons, four protons, and one molecule of oxygen at OEC. This process is known as the Kok cycle, where each intermediate of the catalyst of OEC is referred as the S*i*-state (where *i* = 0–4) (3). The electron generated by the initial charge separation is transferred to two quinone electron accepters, Q_A_ and Q_B_, following a series of charge separation *via* Chl_D1_ and Pheo_D1_. Finally, Q_B_ accepts two electrons and two protons to form a plastoquinol molecule that is released from its binding site and replaced by an oxidized quinone from the plastoquinone pool.

Among the multiple subunits of PSII, D1 is the most important one because it binds most of the active components of the electron transfer chain and also because it undergoes rapid light-induced turn over to protect PSII from photodamage (4, 5, 6). In higher plants, the D1 protein is encoded by a single *psbA* gene, whereas cyanobacteria usually have multiple forms of the *psbA* gene family (7, 8, 9, 10, 11, 12, 13, 14, 15). In the mesophilic cyanobacterium *Synechocystis* PCC 6803, D1 is encoded by three *psbA* genes, *psbAI*, *AII*, and *AIII*, among which, *psbAII* and *psbAIII* encode an identical protein and are expressed under normal and various stress conditions, whereas *psbAI* encodes a protein different from that of *psbAII* and *psbAIII* and has not been found to express under any growth conditions (16). *Synechococcus* PCC 7942 also has three *psbA* genes encoding two different D1 protein isoforms. The expression of the *psbA* genes are altered depending on several environmental conditions such as high light, UV light, or low temperature. The D1:1 isoform encoded by *psbAI* is replaced by the D1:2 isoform encoded by the *psbAII* and *psbAIII* genes, and the functional differences of the two isoforms have been studied with mutant strains (16, 17, 18, 19, 20, 21). In a thermophilic cyanobacterium *Thermosynechococcus elongatus* (*T. elongatus*), three *psbA* genes (*psbA*_*1*_*–*_*3*_) are also identified, which encode three different D1 isoforms (22). The mature D1 protein contains 344 residues, among which, 21 are differ between PsbA1 and PsbA3, 31 are differ between PsbA1 and PsbA2, and 27 are differ between PsbA2 and PsbA3 (Fig. 1). Among these genes, *psbA*_*1*_ is continuously expressed under normal growth conditions; *psbA*_*3*_ is induced at high light conditions (23, 24, 25), and *psbA*_*2*_ is activated under microaerobic conditions (12).

**Figure 1:**
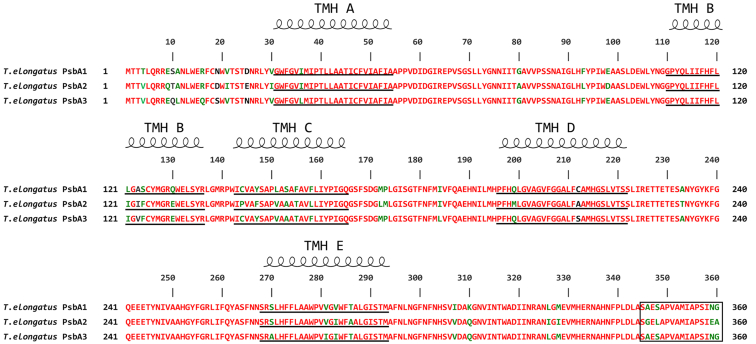
**Amino acid sequence alignment of the three PsbA proteins from *T. elongatus*.** Sequence comparison was performed using Crustal W. The *squared region* in the C terminus indicates sequences that are truncated by posttranslational modification. Residues in *red* indicate the same residues among the three *psbA* genes, residues in *green* indicate two same and one different residues, whereas residues in *black* indicate all different residues among the three genes.

To clarify the functional differences among multiple PsbA proteins in *T. elongatus*, a mutant expressing the *psbA*_*2*_ gene only with the *psbA*_*1*_ and *psbA*_*3*_ genes inactivated (PsbA2 strain) and a mutant expressing the *psbA*_*3*_ gene only with the *psbA*_*1*_ and *psbA*_*2*_ genes inactivated (PsbA3 strain) were reported (26, 27). Spectroscopic studies and crystallographic analyses have shown that the 130th amino acid residue is a glutamine in PsbA1 (PsbA1-Q130), and this residue is hydrogen bonded to the 13^1^-keto group of Pheo_D1_ (1, 28, 29, 30). On the other hand, FTIR spectroscopy measurements suggested that the replacement of PsbA1-Q130 with glutamate (D1-Q130E) in PsbA2 and PsbA3 strains results in a stronger hydrogen bond between PheoD1 and D1-Q130E, thereby altering the redox potential of Pheo_D1_ (30). The redox potential of Pheo_D1_ in PsbA3-PSII was found to increase from −522 mV to −505 mV in PsbA1-PSII (13). Furthermore, the residue at position 270 of the D1 protein is changed from serine in PsbA1 to alanine in PsbA3 (D1-S270A). This change has been suggested to affect the binding of herbicides such as 3-(3,4-dichlorophenyl)-1,1-dimethylurea and bromoxynil (13, 31), hence affecting the binding of the Q_B_ molecule, as this residue is close to the Q_B_ binding site. In PsbA2-PSII, it has been reported that the reduction of P680^+•^ by Tyr_Z_ was slower in the S_2_ and S_3_ states (27). This delay was suggested to be due to changes in proton transfer processes associated with the S-state transitions from both S_2_ to S_3_ and S_3_ to S_0_. In addition, PsbA2 and PsbA3 have many amino acid substitutions relative to PsbA1, which may result in some functional changes such as sensitivity to photodamage, microaerobic condition, etc. (7, 8, 9, 10, 11, 12, 18, 19, 20, 21, 23, 25). Despite these changes, the cell growth rate and oxygen-evolving activity measured under continuous, saturation light did not change significantly; rather, PsbA3-PSII had around twice the oxygen-evolving activity of PsbA1 (26, 27).

In spite of the functional analyses of PSII with different *psbA* genes expressed, whether and how each PsbA protein has an effective function against environmental stress are still unclear. One of the reasons for this is that, unlike PsbA1-PSII, the structures of PsbA2- and PsbA3-containing PSII have not been analyzed, and thus, the results of functional analysis cannot be related with detailed structural information. In this work, we isolated and purified the PSII dimer complexes from mutants deleted of either *psbA*_*1*_ and *psbA*_*3*_ genes (PsbA2 strain) or *psbA*_*1*_ and *psbA*_*2*_ genes (PsbA3 strain) in *T. elongatus*, crystallized them, and analyzed their structures at high resolutions. This provided the structural basis for analyzing their functions under specific stress conditions.

## Results

### Overall structures of PsbA2- and PsbA3-PSII

**Figure 2:**
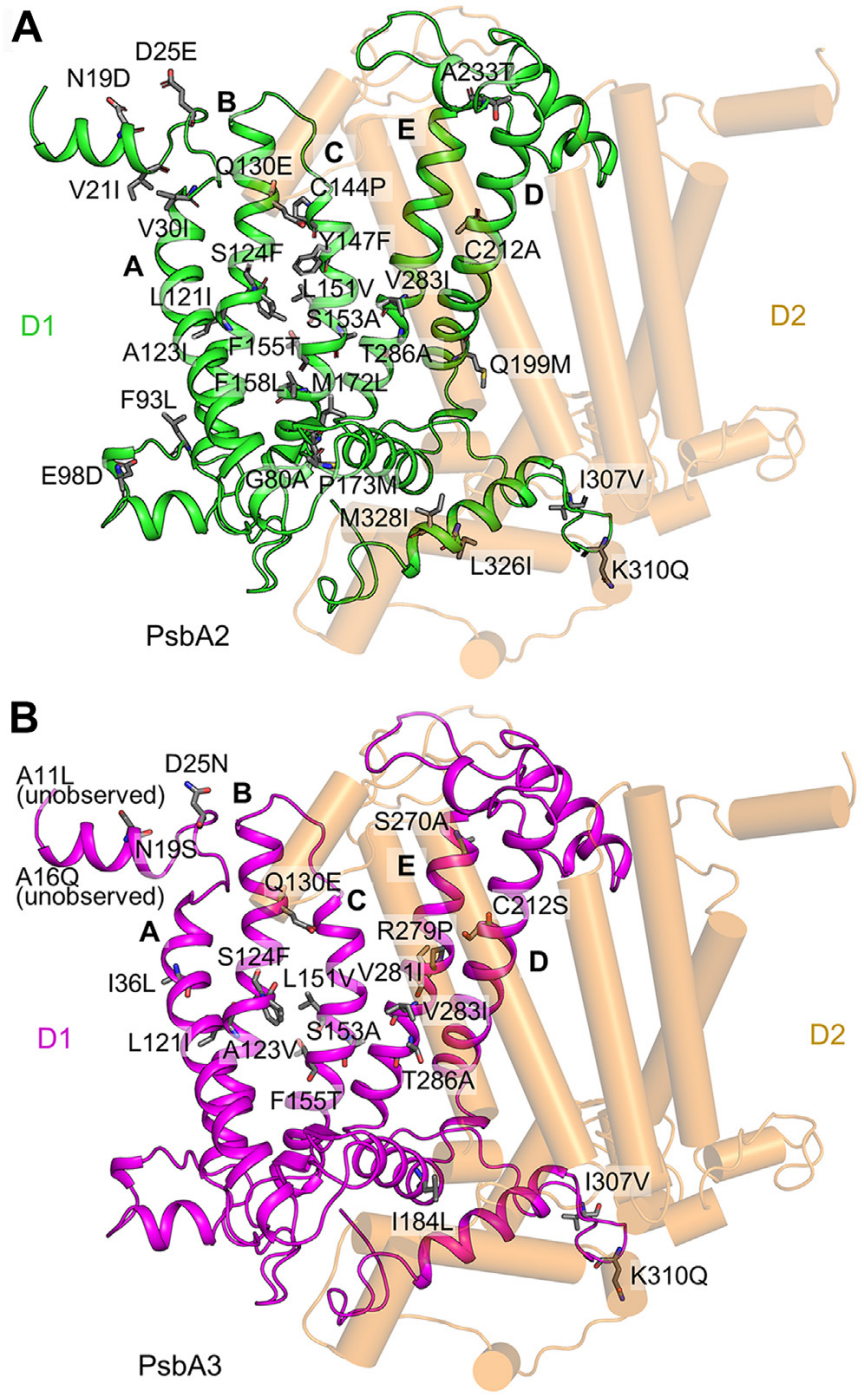
**Overall structure of the D1 and D2 proteins of PsbA2and PsbA3-PSII**. *Green* and *magenta* represent PsbA2 (*A*) and PsbA3 (*B*), respectively, and *beige* represents the D2 protein in both structures. In both panels, (A–E) indicate the transmembrane helices of D1. The side chains of amino acid residues different from those of PsbA1 are shown in *gray*.

Both crystal structures of PsbA2- and PsbA3-PSII dimers were analyzed at a resolution of 1.9 Å (Table 1). Compared to PsbA1-PSII from *Thermosynechococcus vulcanus*, all of the 28 amino acid changes were observed in the PsbA2-PSII structure (Fig. 2*A*). On the other hand, 19 out of the 21 amino acid changes were observed in PsbA3-PSII, and the peripheral region was partially obscured (Fig. 2*B*), which hampered identification of two residues. The RMSD is 0.27 Å for 5240 Cα atoms between PsbA1- and PsbA2-PSII, 0.25 Å for 5201 Cα atoms between PsbA1- and PsbA3-PSII, and 0.20 Å for 5233 Cα atoms between PsbA2- and PsbA3-PSII. For comparison, the RMSD between PSII structures analyzed by synchrotron (Protein Data Bank [PDB] code: 3WU2) and X-ray free-electron laser (PDB code: 4UB6) was 0.33 Å for 5242 Cα atoms (1, 2), which indicates that the overall structure of PSII between the three D1 variants is very similar, and the multiple amino acid changes in PsbA2- and PsbA3-PSII did not have a significant effect on the overall conformation of PSII. This result is consistent with the fact that both strains showed similar growth and oxygen-evolving activity as that of the PsbA1-strain, and the PsbA3-strain even had a higher oxygen-evolving activity than that of the PsbA1 strain (26, 27).

**Table 1:** Data collection and refinement statistics of the datasets collected from PsbA2- and PsbA3-PSII crystals

Strain	PsbA2	PsbA3
Data collection statistics		
Wavelength (Å)	1.0	0.9
Space group	*P*2_1_2_1_2_1_	*P*2_1_2_1_2_1_
Unit cell (Å)	a = 122.1, b = 228.2, c = 286.7	a = 122.8, b = 228.5, c = 286.9
Resolution (Å)	50.0–1.90 (2.01–1.90)^a^	50.0–1.90 (2.01–1.90)^a^
Observed reflections	3,040,947 (473,760)^a^	4,275,973 (687,372)^a^
Unique reflections	622,178 (99,636)^a^	628,112 (100,740)^a^
Completeness (%)	99.8 (99.5)^a^	99.6 (99.4)^a^
Redundancy	4.9 (4.8)^a^	6.8 (6.8)^a^
R_*merge*_ (%)	8.9 (120.0)^a^	9.0 (121.2)^a^
R_*pim*_ (%)	4.4 (66.5)^a^	3.3 (54.7)^a^
CC_1/2_	0.998 (0.689)^a^	0.999 (0.735)^a^
Mean I/σ (I)	11.1 (1.24)^a^	15.8 (1.63)^a^
Refinement statistics		
Resolution (Å)	20.0–1.90	20.0–1.90
R_*work*_/R_*free*_	0.152/0.184	0.147/0.177
Wilson B (Å^2^)	32.1	32.9
R.M.S. deviations		
Bond length (Å)	0.008	0.008
Bong angle (^o^)	1.22	1.28
Ramachandran plot (%)^b^		
Favored	98.05	98.20
Allowed	1.84	1.67
Outliers	0.11	0.13

a Values in parentheses indicate those of the highest resolution shells.

b Ramachandran plot was calculated with MolProbity.

### Structural comparisons of transmembrane helix C between different D1 subunits

**Figure 3:**
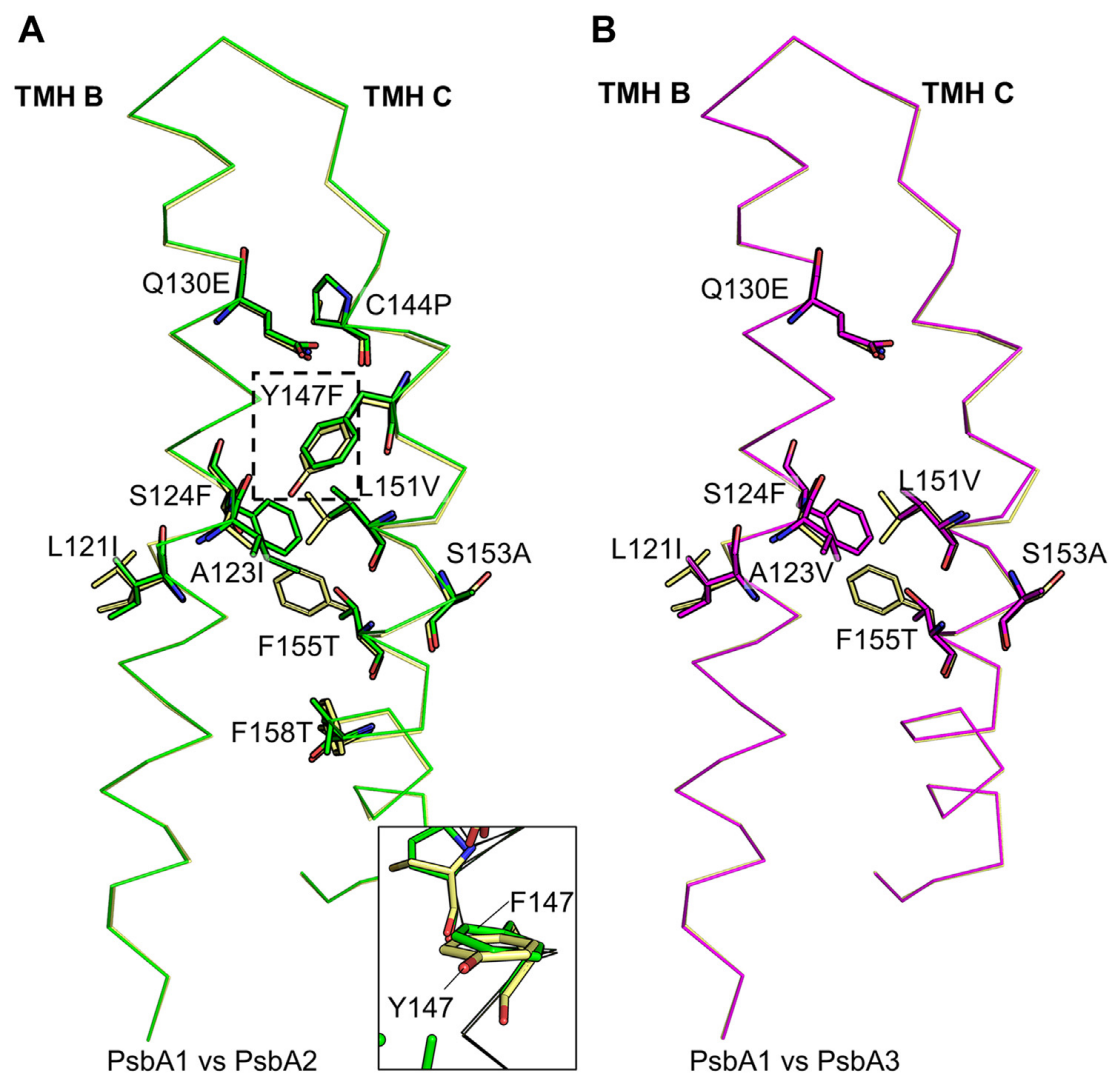
**Comparisons of the backbones and amino acid structures of TMH B and C**. (*A*) Comparison between PsbA1 and PsbA2-PSII. (*B*) Comparison between PsbA1 and PsbA3. TMH B and C of PsbA2-PSII (*green*) and PsbA3-PSII (*magenta*) are superimposed with those of PsbA1-PSII (*yellow*) in panels (*A*) and (*B*), respectively. The *square box* with a *dotted line* in panel (*A*) is enlarged in the *bottom* to show the side chain of D1-147 from a different angle. TMH, transmembrane helix.

In the structure of PsbA2, 6 out of the 28 different residues are located in the transmembrane helix (TMH) C, which starts at Ile143 and ends at Gln165. Importantly, a cysteine residue at position 144 of PsbA1 is replaced by a proline (D1-C144P) in PsbA2 but not in PsbA3 (Figs. 1 and 2). These changes are expected to affect the local structure of TMH C. To reveal the structural changes of TMH C in PsbA2 and PsbA3, the structures of PsbA1, PsbA2, and PsbA3 were superimposed and compared (Fig. 3). The overall structure of the helix is very similar among the three PsbA variants, with RMSDs of the 23 Cα atoms of TMH C between PsbA1 and PsbA2 being 0.23 Å and that between PsbA1 and PsbA3 being 0.21. However, the D1-C144P replacement slightly distorted the main chain structure of TMH C near D1-144 in PsbA2 (Fig. 3), and the change of Tyr147 in PsbA1 to a Phe residue in PsbA2 (D1-Y147F) affected the structure of the side chain itself slightly but did not disrupt the surrounding main chain structure significantly due to the similar side chain sizes between the two subunits (Fig. 3*A*). In addition, the interaction between D1-Phe124 of TMH B and D1-Thr155 of TMH C in both PsbA2 and PsbA3 is swapped compared with the interaction between D1-Ser124 and D1-Phe155 in PsbA1 (Fig. 3, *A* and *B*), due to the (similar) swap of the two residues. These changes make the overall structure of TMH Cs similar among the three PsbA subunits. Owing to these similarities, the conformation of Tyr_Z_, which is located immediately ahead of TMH C, was also similar among the three variants, and thus, the hydrogen bond distance between TyrZ and D1-His190 was not much affected.

### Hydrogen bond environment around pheophytin D1 in PsbA2 and PsbA3

**Figure 4:**
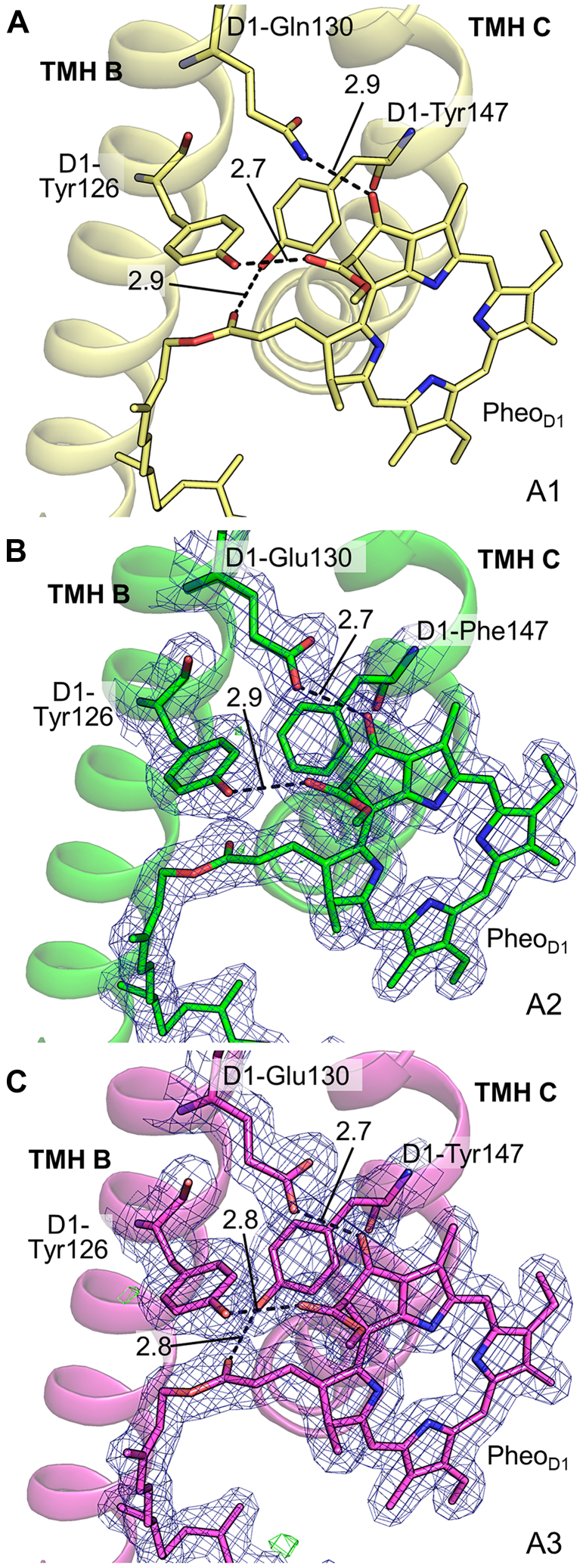
**Comparison of the hydrogen bond environment around Pheo**_**D1**_**among the three D1 variants.** Cartoon and stick models are drawn in *yellow*, *green* and *magenta* for PsbA1 (3wu2) (*A*), PsbA2 (*B*) and PsbA3 (*C*), respectively. The *dotted lines* indicate hydrogen bonds, and the numbers indicate the averaged bond distances between the A- and B-monomers within a dimer. *Blue* and *green meshes* indicate the 2mFo-DFc and positive mFo-DFc density map contoured at 1.0 and 3.5 σ, respectively, in (*B*) and (*C*).

It is known that D1-Tyr126, D1-Glu130, and D1-Tyr147 form hydrogen-bonds with Pheo_D1_ in the structure of PsbA1-PSII (Fig. 4*A*). Among these residues, glutamine at position 130 of PsbA1 is changed to glutamate (D1-Q130E) in both PsbA2 and PsbA3, and the amino acid at PsbA1-Y147 is changed to phenylalanine only in PsbA2-PSII as mentioned previously (Figs. 3*A* and 4). The hydrogen bond distance between D1-E130 and the 13^1^-keto group of Pheo_D1_ in both PsbA2 and PsbA3 strains was shorter than that between D1-Q130 and Pheo_D1_ in PsbA1 by 0.16 to 0.17 Å. This indicates an enhanced hydrogen bond between D1-130 and the 13-keto group of Pheo_D1_, which would cause a stabilization of Pheo_D1_ and hence an increase in its redox potential. This is in good agreement with previous FTIR measurements showing that the redox potential of Pheo_D1_ is increased by 17 mV in the PsbA3-strain than that of the PsbA1-strain (13). However, this increase is found to be half of the redox potential change in a Q130E mutant of *Synechocystis* PCC 6803 (32, 33). This has led to a proposal that other changes in the structure of PsbA3 compensated for the positive shift of the redox potential of Pheo_D1_. Our structure does not reveal significant changes other than the hydrogen bond between Q130E and Pheo_D1_ between PsbA1- and PsbA3-PSII, so the cause for the larger shift in the redox potential of Pheo_D1_ caused by mutation of Gln to Glu in *Synechocystis* 6803 is not clear. On the other hand, the hydrogen bond between D1-Y147 in PsbA1/PsbA3 and the ester group of Pheo_D1_ disappeared in PsbA2 due to its replacement by a nonpolar residue Phe (Figs. 3*A* and 4*B*), making Pheo_D1_ of PsbA2 one hydrogen bond less than Pheo_D1_ of PsbA1/PsbA3. This may suggest that Pheo_D1_ in PsbA2 is the most structurally unstable one among the three D1 variants. However, the redox potential of Pheo_D1_ in PsbA2-PSII has not been reported, and this notation needs to be verified further.

### Influence on hydrogen bond network around OEC in PsbA2

**Figure 5:**
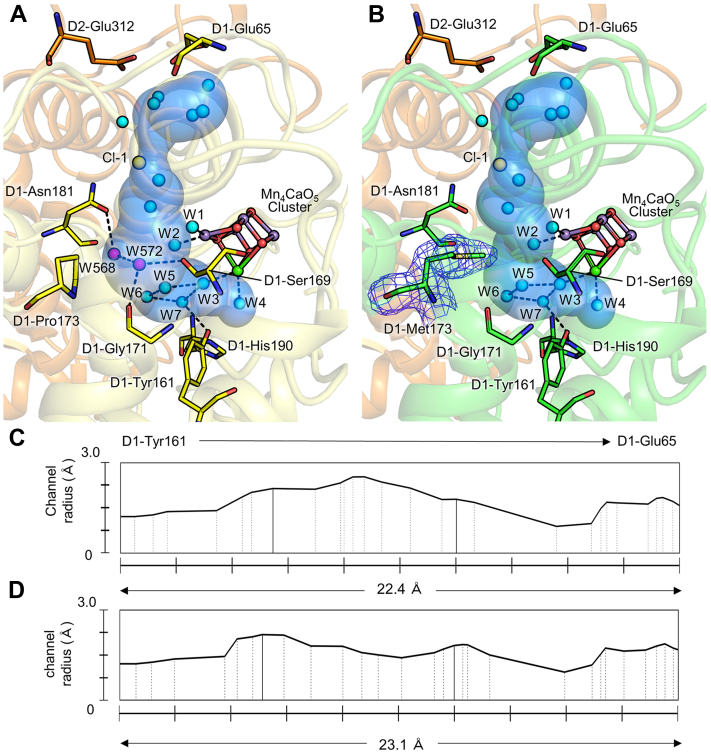
**Comparison of the structures around the Mn**_**4**_**O**_**5**_**Ca cluster in PsbA1and PsbA2-PSII**. (*A*) Structure around the Mn_4_CaO_5_ cluster in PsbA1. (*B*) Structure around the Mn_4_CaO_5_ cluster in PsbA2. The *blue surface* models in (*A*) and (*B*) show the cavity of the Cl-1 channel as calculated by the MOLE program. *Blue mesh* indicates the 2mFo-DFc map contoured at 1.0 σ. (*C*) and (*D*) show the distribution of the channel radii of the Cl-1 channel from the position of D1-Tyr161 to the lumenal surface at D1-Glu65 in PsbA1 (*C*) and PsbA2 (*D*), respectively. In (*C*) and (*D*), the area surrounded by two solid lines indicates the channel region where the residue at position 173 is involved in the composition. Water molecules, Cl, Mn, Ca, O atoms, and the lost water molecules of PsbA2 are colored *cyan*, *yellow*, *purple*, *green*, *red*, and *magenta*, respectively.

The structure of PsbA1-PSII (3WU2) shows several channels from the Mn_4_O_5_Ca cluster to the lumenal solution (1, 2, 4, 5, 34, 35, 36, 37). One of them proceeds toward the bulk surface of PSII *via* the Cl-1 ion and D1-Glu65/D2-Glu312 pair and is named as E65/E312 channel in ref. (37) or Cl-1 channel in ref. (38, 39, 40) or broad channel in ref. (36, 41, 42). We designate this channel as “Cl-1 channel” in this article. Two water molecules (W568 and W572, 3WU2) fill the bulk region inside this channel; they are located around 4.0 Å away from W2. Among these two water molecules, W568 is hydrogen bonded to D1-Asn181, and W572 is hydrogen bonded to the main chain of D1-S169 and D1-G171 in PsbA1-PSII (Fig. 5*A*). In PsbA2-PSII, D1-P173 is replaced with a Met residue (D1-P173M). Due to the larger side chain of Met, the two water molecules cannot stay in their original positions, so they became invisible (Fig. 5*B*), whereas they are not changed in PsbA3-PSII due to the same residue of Pro in position 173 (not shown). The radius of the Cl-1 channel of PsbA2 calculated showed that it is narrowed due to the larger side chain of Met (Fig. 5, *C* and *D*). The average diameter of the region where D1-173 is involved is around 0.9 Å narrower in PsbA2-PSII than that of the PsbA1-PSII, and the narrowest region is around 2.8 Å in PsbA2-PSII. This may lead to changes in the distribution of water molecules and hydrogen bond patterns, which may limit the proton egress *via* a Grotthus-type transfer process. Indeed, a previous study has shown that the reduction of P680^+•^ by Tyr_Z_ was much slower in PsbA2-PSII in the S_2_- and S_3_-states, but not in the S_1_-state, compared with that in PsbA1- and PsbA3-PSII (27, 43). This has been suggested that in the S_2_- and S_3_-states the increased positive charge could weaken the strength of the hydrogen bond interaction between Tyr_Z_^•^ and D1-His190 in PsbA2 *versus* PsbA3, and/or the D1-P173M change may induce structural modification(s) of the water molecule network around Tyr_Z_, resulting in the slow proton egress and therefore the slower oxidation of P680^+•^ by Tyr_Z_. Our present results showed that the structure of the Mn_4_CaO_5_ cluster (Table S1), the arrangement of water molecules, and the hydrogen bonding environment around Tyr_Z_ are very similar among the three PsbA variants, except the narrowing of the proton path in the PsbA2 strain as mentioned previously (43). Thus, the slower oxidation of P680^+•^ by Tyr_Z_ in higher S-states in the PsbA2 strain may be caused by the limitation in the proton egress channel due to the D1-P173M change.

### Changes in the environment of P680 among the three PsbA variants

**Figure 6:**
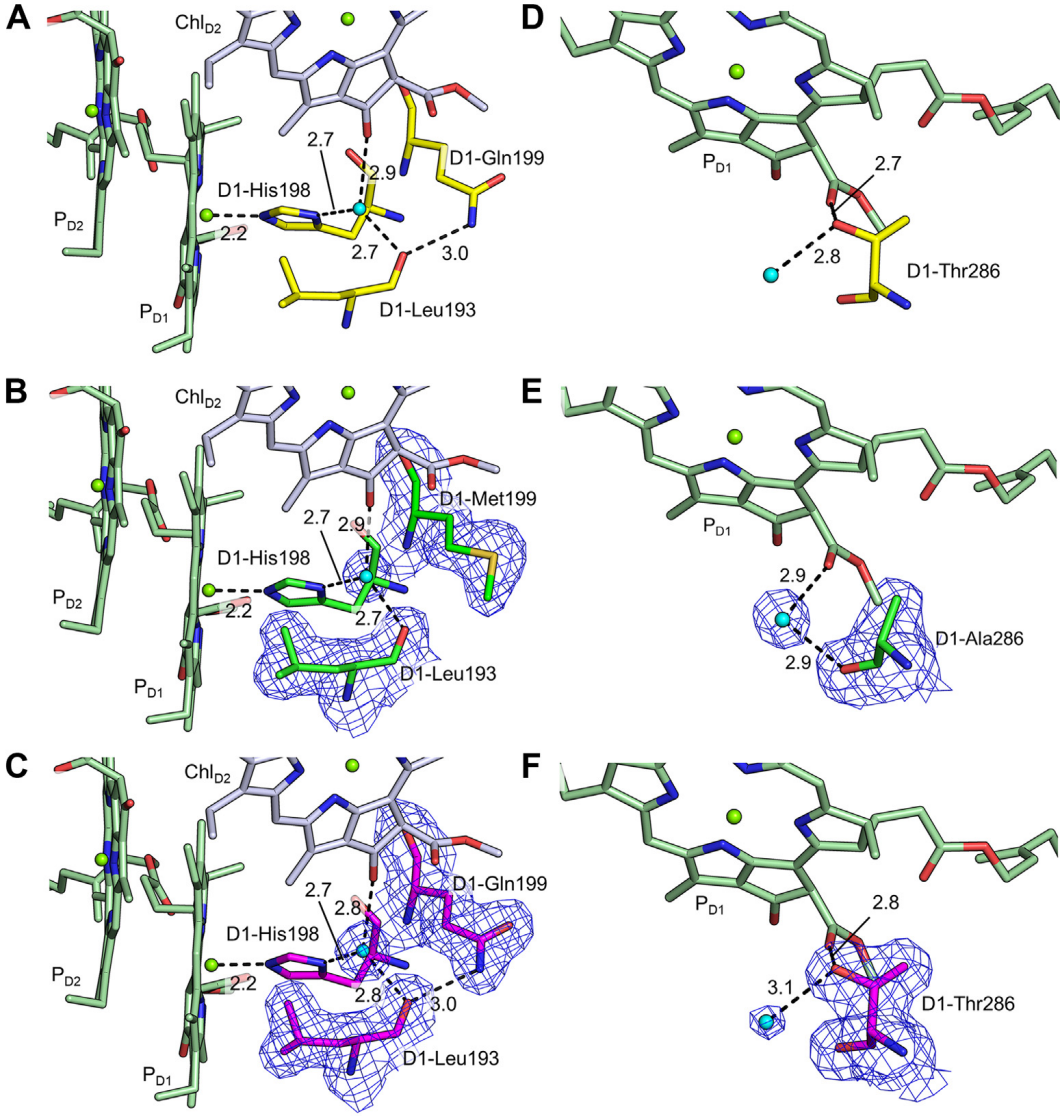
**Comparison of hydrogen bonding environment around P**_**D1**_**(P680).***A*–*C*, show the hydrogen bonding environment in PsbA1 (pdb code: 3WU2), PsbA2, and PsbA3, respectively, and (*D*–*F*) show the hydrogen bonding environment when viewed from different angles in each of (*A*–*C*). The *black dotted lines* represent hydrogen bonds, and the numbers show the hydrogen bond distances (Å) averaged between A- and B-monomers within a dimer. *Cyan* spheres indicate water molecules. The *blue mesh* shows the 2mFo-DFc map contoured at 1.0 σ.

In PsbA1 and PsbA3, Gln199 and Thr286 are surrounding P_680_, whereas in PsbA2, these two residues are changed to Met199 and Ala286. Gln199 is hydrogen bonded to Leu193, which in turn is hydrogen bonded to both Chl_D2_ and His198 through a water molecule, the latter being a direct ligand to P_D1_ (Fig. 6). In PsbA2, the hydrogen bonds of P_D1_ and Chl_D2_ are not changed, but the hydrogen bond between the main chain of Leu193 and Gln199 disappeared due to the replacement by Met199. This may lead to a slight instability of P_D1_ and/or Chl_D2_ in PsbA2. In addition, Thr286 is directly hydrogen bonded to P_D1_ in PsbA1 and PsbA3, whereas Ala286 in PsbA2 cannot hydrogen bond to P_D1_ and instead a water molecule is directly hydrogen bonded to P_D1_. This may also reduce the stability of P_D1_ and contribute to the slower oxidation of P680^+•^ by Tyr_Z_ in higher S-states in the PsbA2 strain as mentioned previously.

### Structural comparison around the Q_B_ binding site between PsbA1 and PsbA3

**Figure 7:**
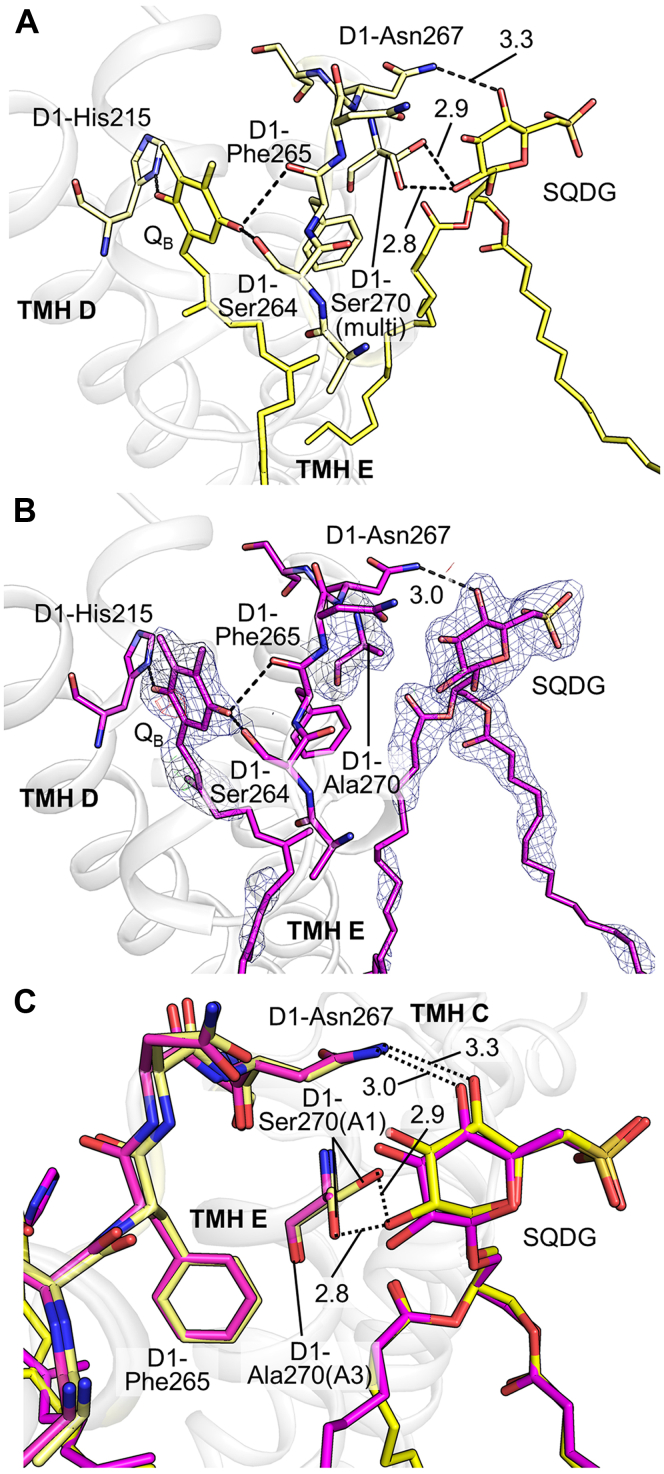
**Structural comparison of PsbA1and PsbA3-PSIIaround the Q**_**B**_**and SQDG binding regions.** (*A*) Structure of PsbA1. (*B*) Structure of PsbA3. *Dotted lines* indicate hydrogen bonds, and the numbers show the averaged bond distances between A- and B-monomers within a dimer (Å). Only Q_B_, SQDG, their hydrogen bonding partners, and the amino acid residues that make up the Q_B_ loop were illustrated with stick model for clarity and colored in *yellow* for PsbA1 and *magenta* for PsbA3, in (*A*–*C*). (*C*) is the superimposed structure of PsbA1 and PsbA3 around the Q_B_ cavity and SQDG. The *black dotted lines* represent the hydrogen bonds, and the numbers show averaged hydrogen bond distances (Å) between A- and B-monomers within a dimer. The *blue mesh* shows the 2mFo-DFc map contoured at 1.0 σ. SQDG, sulfoquinovosyl diacylglycerol.

D1-S270 adopts two conformations in PsbA1 and PsbA2, and both conformations are hydrogen bonded to the sulfoquinovosyl diacylglycerol (SQDG) in PsbA1 and PsbA2-PSII (Fig. 7*A*). This hydrogen bond was lost in PsbA3-PSII due to the change of D1-S270 to Ala (Fig. 7*B*). SQDG that has lost one of the hydrogen bonding partners in PsbA3 has a closer distance to D1-Asn267 than those of PsbA1 and PsbA2, as a result of slight shift of the SQDG head group (Fig 7, *B* and *C*). This resulted in an average hydrogen bond distance between A- and B-monomers between SQDG and D1-Asn267 of PsbA3 that was 0.3 Å shorter than that in PsbA1 and 0.4 Å shorter than that in PsbA2. The main chain nitrogen of Phe265 is hydrogen bonded to the carbonyl group of the Q_B_ head region, which was 0.2 Å shorter in PsbA2 and PsbA3 than that in PsbA1, respectively. D1-Ser264 is also hydrogen bonded to Q_B_, and its distance is 0.2 to 0.3 Å shorter in PsbA2 and PsbA3 than that in PsbA1. Furthermore, the B-factor of D1-Ser264 decreased to 51.8 Å^2^ for PsbA3 compared to 59.8 Å^2^ for PsbA1 and 60.1 Å^2^ for PsbA2. The B-factor of the Q_B_ head region also decreased slightly in PsbA3-PSII compared with those in PsbA1- and PsbA2-PSII, resulting in a more clearly defined density map of the Q_B_ head region of PsbA3-PSII compared with those of PsbA1 and PsbA2 (Fig. S1). All these results suggested a more stable binding of Q_B_ to its binding site in PsbA3 than those in PsbA1 and PsbA2.

## Discussion

We have succeeded in analyzing the crystal structures of PsbA2- and PsbA3-PSII dimers at their dark-stable state (S_1_-state) with a resolution comparable to that of PsbA1-PSII. The results confirmed the amino acid changes in both D1 variants. However, the hydrogen bond distance between the 13^1^-keto group of Pheo_D1_ and D1-E130 was shortened in both PsbA2 and PsbA3 due to the change of Gln to Glu, in agreement with the result of FTIR analysis (30) showing the enhancement of interactions between Pheo_D1_ and D1-E130 in PsbA2 and PsbA3. On the other hand, a hydrogen bond of Pheo_D1_ was lost due to the D1-Y147F substitution in PsbA2, suggesting that the structural stability of Pheo_D1_ is decreased in PsbA2. The breakage of the hydrogen bond between D1-F147 and Pheo_D1_ may be necessary to avoid conformational changes of TMH C caused by amino acid changes of D1-144, where it is a Cys in both PsbA1 and PsbA3 but a Pro in PsbA2. This change caused a disruption in the TMH C main chain, which subsequently caused a flip of D1-F147. As a result, the structure of the TMH C main chain is kept rather constant. In addition, the D1 sequence alignment of various species focusing on the combination of amino acids between positions 144 and 147 (Fig. S2) shows that the majority of the species in which the amino acid corresponding to position 144 in *T. elongatus* is proline have Phe at the position 147. These amino acid alignment and structural analyses suggest that changes in the orientation of the TMH C due to the expression of proline may be disadvantageous for survival when the effect is transmitted to Pheo_D1_ *via* amino acid at positions D1-147. To confirm this, it is necessary to examine cell growth, redox potential of Pheo_D1_, and the crystal structure of PSII in a mutant strain that simultaneously expresses Pro at D1-144 and Tyr at D1-147.

The amino acid residue in the position 173 of the D1 subunit is changed from proline in PsbA1 and PsbA3 to methionine in PsbA2. We showed that this change caused the disappearance of two water molecules and narrowing of the Cl-1 channel due to invasion of the side chain of methionine in the PsbA2-PSII structure. In the previous report, it has been suggested that the Cl-1 channel is a reasonable candidate for the intake of water molecules from the bulk surface of PSII (34, 37). The structural analyses showed that water molecules such as W3, 4, 5, 6, which are located deeper than the narrowed channel region (44), are present in PsbA2-PSII similar as those in PsbA1 and PsbA3. Thus, if the Cl-1 channel is responsible for the water uptake, the narrowing of the Cl-1 channel observed in PsbA2-PSII would have no significant effect or other channels may be utilized for the water uptake. Time-resolved absorption spectroscopy showed a marked delay in the reduction of P680^+^ by Tyr_Z_ after two and three flashes in PsbA2, and similar features were reported in the site-specific mutant strain of PsbA3 with D1-P173 replaced by a methionine (43). Considering these spectroscopic results and the report that the Cl-1 channel is the main pathway for proton transfer in the S_3_->S_0_ state transition, it is suggested that the loss of water molecules by D1-P173M and the narrowing of the Cl-1 channel may affect proton transfer and consequently cause a delay in the reduction of P680^+^ in the S_2_ and/or S_3_ states (39, 45, 46, 47, 48, 49, 50, 51). Considering the location of the disappeared water molecules, there may also be a connection between these waters and the Yz network. It has been reported that proton-coupled electron transfer through the Y_Z_ network consisting of Tyr_Z_ and surrounding water molecules is dominant in the S_2_->S_3_ state transition (52). Nakamura *et al.* (53) proposed a model in which protons generated near O5 in the Mn_4_O_5_Ca cluster are transferred to Tyr_Z_ through multiple pathways in the Yz network. In the detour route, which is not the shortest route to Tyr_Z_, there is a W5 that is hydrogen bondable to the water molecule (W572) excluded by the D1-P173M exchange (2, 40, 54). It is assumed that the loss of this water molecule will result in the loss of hydrogen bonds of W5, which affect proton transfer in the bypass route involving W5. The two water molecules may therefore contribute to the highly efficient proton transfer in PsbA1 and PsbA3 but lost in PsbA2-PSII, resulting in a lower efficiency of proton transfer.

The only amino acid change that occurred near the Q_B_-binding region is D1-270, which is a Ser in PsbA1 and PsbA2 but Ala in PsbA3. This resulted in the cleavage of the hydrogen bond between this residue and the SQDG molecule bound near the Q_B_-binding region. This resulted in a change in the binding state of the SQDG head region and an increased binding strength to D1-Asn267 in PsbA3. In the exchange process of Q_B_ molecule, molecular dynamics simulations indicated that the conformational changes of the Q_B_ loop region provide the driving force for the movement of the Q_B_H_2_ headgroup (55). In addition, crystallographic analysis of the S_3_ intermediate state using a free-electron laser demonstrated that the loop region from D1-Asn266 to Ser268 moves by up to 0.8 Å upon reduction of Q_B_, resulting in the partial opening of the Q_B_-binding site (54). These reports suggest that the mobility of the Q_B_ loop greatly affects the Q_B_ exchange process. Therefore, changes in the hydrogen bonding environment of amino acids involved in the movement of the loop should affect the efficiency of Q_B_ exchange. In addition to the mobility of the entire loop, the changes in amino acid residues around the Q_B_ head region that occurred in PsbA3 may have a direct effect on the Q_B_ exchange process. Previous theoretical studies have proposed that the hydroxyl group of D1-Ser264 is oriented toward the carbonyl group of Q_B_ by proton uptake by D1-His252, which stabilizes the head region of Q_B_ and facilitates the initial electron transfer from Q_A_ to Q_B_ (56). Thus, the proximity of the main chain of D1-Phe265 to Q_B_ and the stabilization of Ser264 may contribute to stabilize the head region of Q_B_ and to improve the efficiency of electron transfer to Q_B_ in PsbA3. These changes in the environment around the Q_B_-binding region may also affect the binding of the inhibitors that have been reported (10, 31). These indirect effects derived from SQDG-binding status on Q_B_ exchange were also reported in the crystal structure and functional analysis of PSII from a SQDG-deficient strain (57). To clarify how the change in the SQDG-binding state affects the movement of the loop region surrounding Q_B_ and Q_B_ molecule during the exchange process, it will be necessary to analyze the intermediate structures of the S_2_ and S_3_ states in PsbA3-PSII.

In conclusion, we obtained high-resolution structural information of PsbA2- and PsbA3-PSII from strains that express PsbA2 or PsbA3 only. The overall structure of PSII is highly conserved between PsbA1, PsbA2, and PsbA3, implying the importance of the D1 protein in PSII reactions. However, the amino acid changes seen in the three *psb*A genes affected the properties of Pheo_D1_, the S-state transition efficiency, and the Q_B_-binding properties. A part of these effects is caused by the changes of some water molecules and lipids, whose roles have not been defined clearly so far. The present crystal structural analyses thus provide an important structural basis for future mutagenesis and simulation studies on the functions of the PsbA variants.

## Experimental procedures

### Cell culture of D1 variants and purification of PSII dimers

Strains of PsbA2-PSII (lacking *psbA*_*1*_ and *psbA*_*3*_, *ΔpsbA*_*1*_*ΔpsbA*_*3*_) (27) and PsbA3-PSII (lacking *psbA*_*1*_ and *psbA*_*2*_, *ΔpsbA*_*1*_*ΔpsbA*_*2*_) were constructed as described previously (26). The PsbA2 strain was cultured in the presence of 5 μg/ml chloramphenicol, 25 μg/ml spectinomycin, and 10 μg/ml streptomycin, and the PsbA3 strain was cultured in the presence of 5 μg/ml chloramphenicol, in 2 l with constant red LED illumination at an intensity of 20 μmol photons m^−2^ s^−1^. After the cells were grown to their logarithmic phase, they were diluted to 40 l and cultured in the absence of antibiotics with gradual increase of light intensity from 40 to 100 μmol photons m^−2^ s^−1^ for 7 to 8 days. The cells harvested were broken and PSII dimers were purified using methods reported previously with slight modifications (57, 58). For solubilization of the thylakoid membranes from both mutants, 0.25% (v/v) lauryl dimethylamine-n-oxide was utilized, which was followed by solubilization with n-dodecyl-β-D-maltoside and purification of the PSII dimers with an anion-exchange column chromatography.

### Crystallization and structural analysis

The PsbA2- and PsbA3-PSII dimers purified were crystallized using an oil batch method and recrystallized under conditions described previously (57, 58). In both PSII mutants, crystals grew to a size of around 1.0 × 0.4 × 0.2 mm, and they were collected and treated by cryoprotectant solution with buffer conditions reported previously (57, 58). The crystals were then flash-frozen in a nitrogen gas stream at 100 K. X-ray diffraction images were collected at the beamline BL41XU of SPring-8, Japan. The dataset obtained was indexed, integrated, and scaled with XDS (59). The initial phase up to 4 Å resolution was obtained by molecular replacement with PaserMR in CCP4 program suite (60) using the 1.90 Å resolution structure of native PSII (PDB accession code: 3WU2) as the search model, and both structures were refined to 1.90 Å resolution with Refmac5 of CCP4 program suite (61) and Phenix refinement (62). The RMSD was calculated with lsqkab in the CCP4 program suite (63). Model building was performed with COOT (64), and figures were made with PyMOL (65). The tunnel cavity model was calculated by the program MOLE 2.0 (66).

## Data availability

The structures reported in this article have been deposited in PDB with the accession codes of 7YQ2 for PsbA2-PSII and 7YQ7 for PsbA3-PSII. All other data are available from the authors upon reasonable request.

## Supporting information

This article contains supporting information.

## Conflict of interest

The authors declare that they have no conflicts of interest with the contents of the article.
