# Supplementary material for: Crystal structures of photosystem II from a cyanobacterium expressing psbA2 in comparison to psbA3 reveal differences in the D1 subunit
Source: J Biol Chem. 2022 Nov 2;298(12):102668. doi: 10.1016/j.jbc.2022.102668 (PMC9709244; doi:10.1016/j.jbc.2022.102668)
Supplement: Table S1, Figs. S1 and S2 [file mmc1.docx]

Supporting Information

**Crystal structures of photosystem II from a cyanobacterium expressing *psbA_2_* in comparison to *psbA_3_* reveal differences in the D1 subunit**

Yoshiki Nakajima^1^, Natsumi Ugai-Amo^2^, Naoki Tone^2^, Akiko Nakagawa^3^, Masako Iwai^4^, Masahiko Ikeuchi^4^, Miwa Sugiura^3^, Michihiro Suga^1,2,*^ and Jian-Ren Shen^1,2,*^

*^1^**Research Institute for Interdisciplinary Science, Okayama University, 3-1-1 Tsushima Naka, Okayama 700-8530, Japan*

*^2^Graduate School of Natural Science and Technology, Okayama University, 3-1-1 Tsushima Naka, Okayama 700-8530, Japan*

*^3^Proteo-Science Research Center, Ehime University, Matsuyama 790-8577, Japan*

*^4^Graduate School and College of Arts and Sciences, The University of Tokyo, 3-8-1 Komaba, Meguro-ku, Tokyo 153-8902, Japan*

*Corresponding authors*

*Michihiro Suga, E-mail: msuga@okayama-u.ac.jp*

*Jian-Ren Shen, E-mail: shen@cc.okayama-u.ac.jp*

**Table S1. Inter-atomic distances (Å) within the Mn_4_CaO_5_ cluster of photosystem II among the three different D1 variants.**


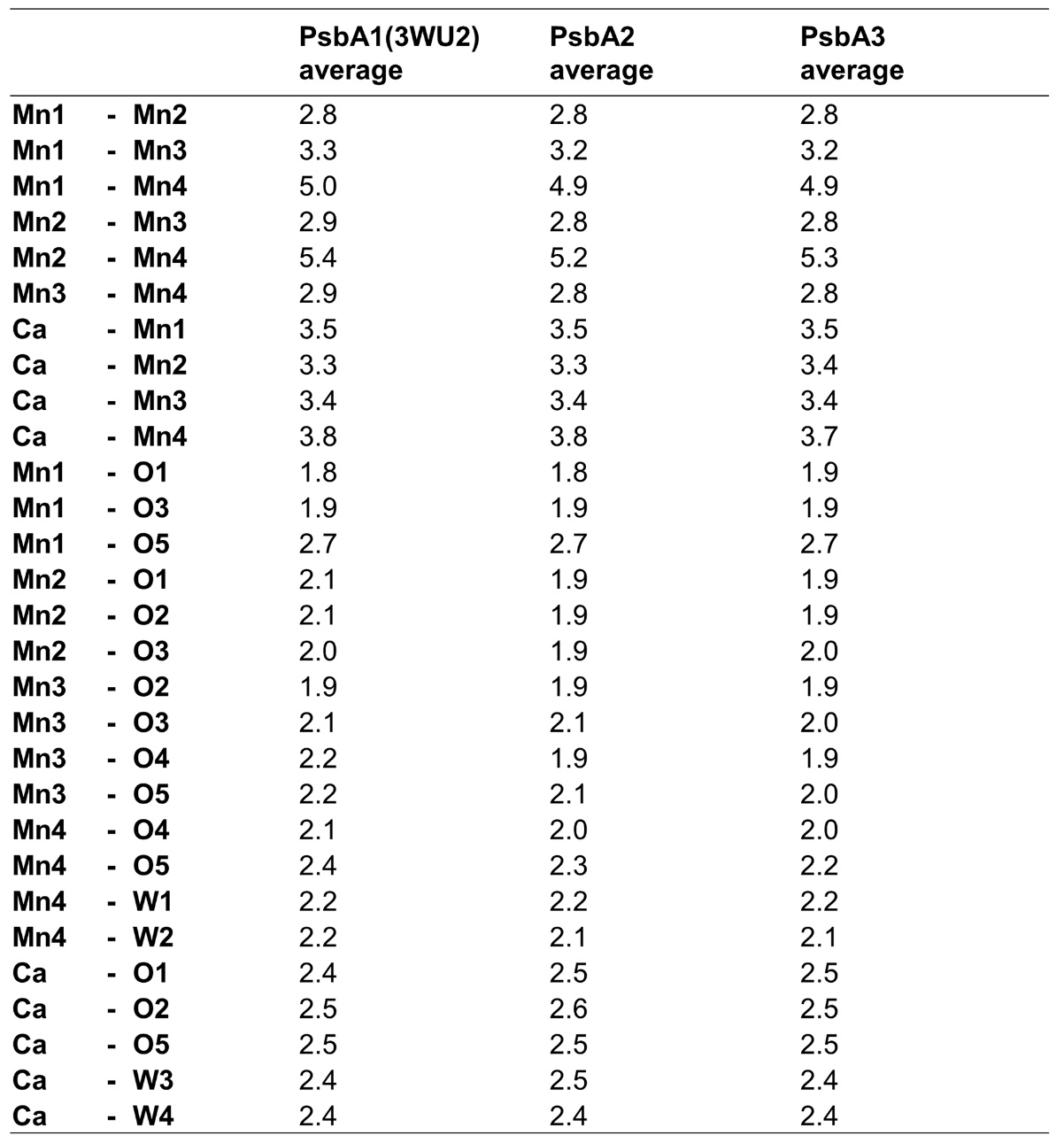


The values are the average lengths between A- and B-monomers within a PSII dimer.


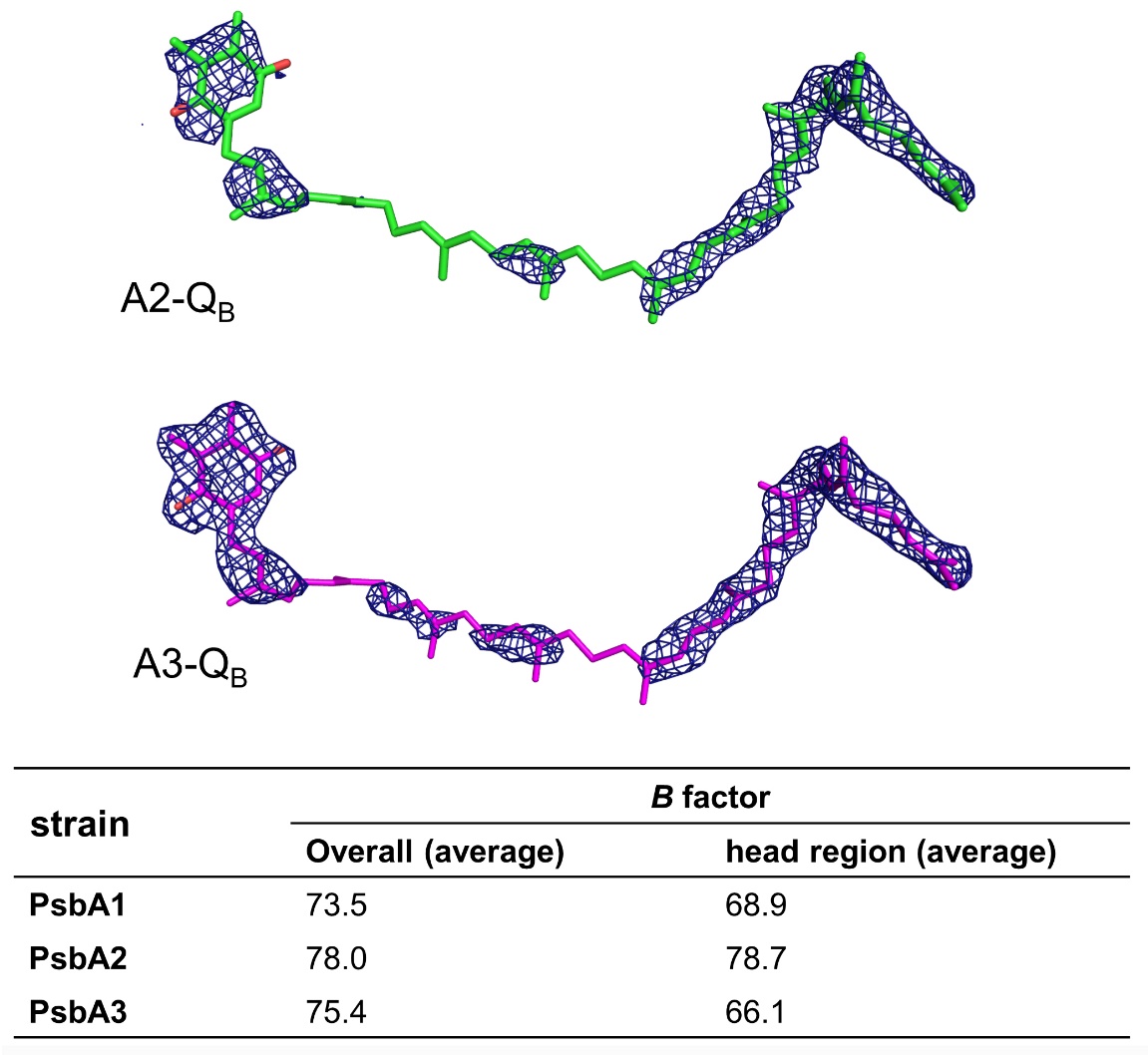


**Fig. S1. Comparison of the electron density maps and averaged B-factors of the Q_B_ molecule between A and B monomers within a dimer.** Blue mesh indicates the 2mFo-DFc map contoured at 1.0 σ. In the lower table, B-factors of the head region including atoms O1, O2 and those from C1 to C6 of the Q_B_ molecule are shown.


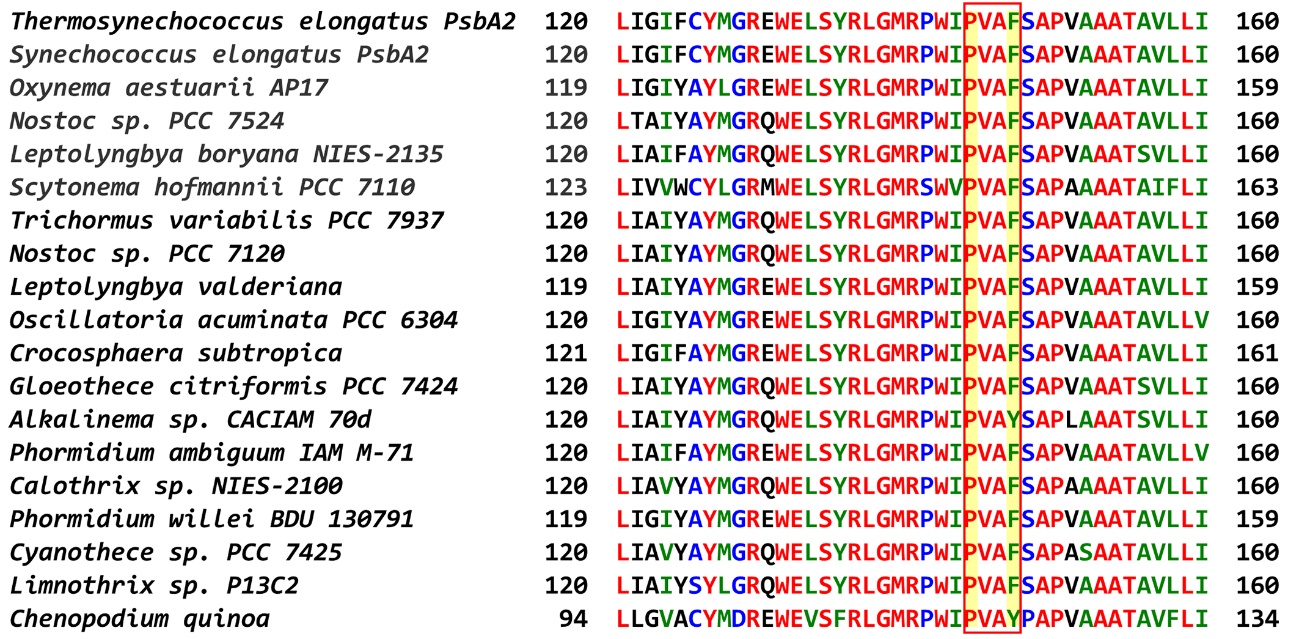


**Fig. S2. Amino acid sequence alignment of the PsbA proteins from various organisms.** Sequence comparison was performed using Crustal W. Sequences with a proline residue at position 144 (corresponding to the PsbA2 protein) were selected, and the regions surround the 144 residue were compared.
